# Continuous Positive Airway Pressure Improves Renal Function in Obese Patients With Obstructive Sleep Apnea Syndrome

**DOI:** 10.3389/fmed.2021.642086

**Published:** 2021-03-03

**Authors:** Maria Perticone, Raffaele Maio, Paola Elisa Scarpino, Luana Mancuso, Mara Volpentesta, Benedetto Caroleo, Edoardo Suraci, Angela Sciacqua, Giorgio Sesti, Francesco Perticone

**Affiliations:** ^1^Department of Medical and Surgical Sciences, Magna Graecia University, Catanzaro, Italy; ^2^Geriatrics Division, Azienda Ospedaliero-Universitaria Mater Domini, Catanzaro, Italy; ^3^Geriatrics Division, Azienda Ospedaliera Annunziata, Cosenza, Italy; ^4^Department of Clinical and Molecular Medicine, La Sapienza University, Rome, Italy

**Keywords:** chronic kidney disease, renal function, OSAS, CPAP, cardiovascular risk factors

## Abstract

**Background:** Obstructive sleep apnea syndrome (OSAS) is an independent risk factor for cardiovascular morbidity and mortality, and it has a detrimental effect on renal function. Obesity is the major risk factor for OSAS, and represents a risk factor for chronic kidney disease. Continuous positive airway pressure (CPAP) is the suggested therapy for moderate-to-severe OSAS. We designed this study to evaluate the effect of CPAP on estimated glomerular filtration rate (e-GFR) in a cohort of obese patients with moderate-to-severe OSAS and normal renal function.

**Methods:** We enrolled 198 obese subjects, divided into two groups (OSAS+ and OSAS–), on the basis of cardiorespiratory monitoring; mild OSAS patients (*n* = 33) were excluded from the study, thus the analyses were conducted on 165 patients. Comparisons between groups were made by Student *t*-test or χ^2^ test as appropriate. Linear regression analyses were used to assess the relationship between baseline e-GFR and different covariates and, in the OSAS+ group, between Δe-GFR and different covariates. A multivariate regression analysis was performed to determinate the independent predictor of the Δe-GFR.

**Results:** OSAS+ subjects showed significantly increased values of systolic blood pressure, HOMA, pulse wave velocity, high-sensitivity C reactive protein and uric acid compared with OSAS– group. OSAS+ group showed significantly lower values of e-GFR and increased values of microalbuminuria. At linear regression analysis e-GFR resulted significantly and inversely related to AHI in the whole study population and in the two groups. After 6 months of CPAP therapy, OSAS+ subjects showed an improvement in respiratory parameters, as well as a significant increase in e-GFR values (104.2 + 19.0 vs. 84.0 + 13.1 ml/min/1.73 m^2^, *P* < 0.0001). At multiple regression analysis, Δ apnea/hypopnea index (AHIa) resulted the main independent predictor of Δe-GFR explaining 22% of its variation.

**Conclusions:** Obese OSAS patients show significantly lower values of e-GFR, even if in the normal range, compared with obese non-OSAS subjects. After 6 months of CPAP, e-GFR significantly improved (+20 ml/min/1.73 m^2^) and ΔAHIa resulted the most important independent predictor of Δe-GFR.

## Introduction

In the last years obstructive sleep apnea syndrome (OSAS) has been considered as an independent risk factor for cardiovascular (CV) morbidity and mortality, similarly to other classical risk factors such as obesity, diabetes mellitus (DM), and hypertension (1). Even if the pathogenetic mechanisms underlying the association between OSAS and CV diseases have been not completely elucidated, hypoxia-induced endothelial dysfunction (2, 3), insulin resistance (4), and sympathetic-related CV hemodynamics (5, 6) seem to play a pivotal role.

Renal function decline, evaluated by estimated glomerular filtration rate (e-GFR), is associated with increased CV morbidity and mortality in the general population as well as in various settings of patients (7–9), and mild-to-moderate renal insufficiency has emerged as a major public and clinical health problem. It has been hypothesized that chronic kidney disease (CKD) shares common pathogenetic mechanisms with OSAS, such as increased oxidative stress, metabolic alterations and sympathetic activation, which could explain the link between OSAS and renal function decline (10). Moreover, secondary analyses of intervention studies conducted in both hypertensive patients (11) and selected patients at high CV risk (12) have shown that classical CV risk factors such as hypertension, DM, hyperlipidemia, smoking, and overweight/obesity represent major correlates of renal dysfunction.

However, the relationship between OSAS and renal function decline needs to be further elucidated; it is well-known that OSAS is highly prevalent among individuals with end-stage renal disease (ESRD) (13) and that patients with early stages of CKD show an increased risk of OSAS compared to subjects with normal renal function (14), but the cause-effect link between these conditions has not yet been clearly explained. In fact, there is also evidence that ESRD may induce a progression of CKD, thus indicating a bidirectional relationship between the conditions (15).

On the other hand, several data showed that obesity, the main risk factor for OSAS, is also associated with dysregulation of renal function, such as glomerular hyperfiltration and increased albuminuria, thus representing a risk factor for CKD and its progression to kidney failure (16–18).

Currently, the gold-standard treatment of moderate-severe OSAS is continuous positive airway pressure (CPAP), regardless of body weight (19). This therapy has shown a reduction/disappearance of nocturnal hypoxic episodes which, in turn, determinates the improvement of several hemodynamic and cardiometabolic parameters.

Previous studies investigating the effect of CPAP treatment on renal function were conducted in patients with CKD (20) or in non-obese OSAS patients with normal renal function (21) but, to our knowledge, no data exist in obese OSAS subjects with normal renal function. Thus, we designed this study to evaluate the effect of CPAP therapy on renal function in a cohort of well-characterized obese patients with moderate-to-severe OSAS and preserved renal function (e-GFR > 60 ml/min/1.73 m^2^).

## Materials and Methods

### Study Population

We recruited 198 obese Caucasian outpatients (body mass index, BMI >30 Kg/m^2^; 110 males and 55 females; mean age 55.3 ± 11.0 years) referred to the Sleep Breathing Disorders Clinic of the University Hospital of Catanzaro, who underwent cardiorespiratory monitoring (CRM) during sleep for reported daytime sleepiness and persistent (>6 months) snoring. None of the patients had history or clinical evidence of angina, myocardial infarction, valvular heart disease, arrhythmias, peripheral vascular disease, arterial hypertension, DM, coagulopathy or any disease predisposing to vasculitis or Raynaud's phenomenon, liver and respiratory disease, lung failure, CKD (defined for e-GFR values <60 ml/min/1.7 m^2^) or urinary tract infections. At the time of the first evaluation, we collected medical history and performed physical examination evaluating body weight, height and BMI, waist and neck circumference (NC), determination of arterial stiffness, routine blood tests, and 75-g oral glucose tolerance test (OGTT) after 12 h fasting, 0 and 120 min of sampling for plasma glucose and insulin determination. According with clinical guidelines for OSAS management (22), all subjects participating to the study received general education about the importance of weight loss, sleep position, alcohol avoidance and risk factors modifications. Subjects who were diagnosed with moderate-to-severe OSAS were prescribed with CPAP therapy. In accordance with the aim of the study, OSAS patients treated with CPAP were specifically re-evaluated for respiratory parameters, renal function and CV risk factors 6 months after the beginning of CPAP therapy; after the observation period, appropriate anti-hypertensive and/or insulin-sensitizers medications were prescribed, when needed. At the end of the follow-up period, all subjects whose BMI remained in the obesity range were prescribed with a hypocaloric diet with a caloric deficit of 500 kcal/die, in order to achieve a BMI of 25 kg/m^2^ or less. The local Ethics Committee (Calabria Centro) approved the protocol (approval number 2,012.63) and informed written consent was obtained from all participants. All the investigations were performed in accordance with the principles of the Declaration of Helsinki.

### Laboratory Determinations

All laboratory measurements were performed after a fast of at least 12 h. Plasma glucose was determined immediately by the glucose oxidase method (Glucose Analyzer, Beckman Coulter S.p.A., Milan, Italy). Triglyceride and total, low-density lipoprotein (LDL), and high-density lipoprotein (HDL) cholesterol concentrations were measured by enzymatic methods (Roche Diagnostics GmbH, Mannheim, Germany). Plasma concentrations of insulin were determined by chemiluminescence test (Roche Diagnostics). Insulin resistance was determined by homeostasis model assessment (HOMA) index, calculated according with the following equation:

$$\begin{array}{l} \text{HOMA}=\text{fasting insulin }(\mu \text{U/mL})\;\times \text{ fasting glucose }(\text{mmol/L})\\ \;/22.5 \end{array}$$

(23)

that is strongly related to the euglycemic clamp, which represents the gold standard test for measuring insulin sensitivity. Creatinine measurements and uric acid (UA) were performed at baseline by use of the Jaffe methodology and the uricase peroxidase (uricase/POD; Boehringer Mannheim, Mannheim, Germany) method implemented in an autoanalyzer. Values of e-GFR (mL/min/1.73 m^2^) were calculated with the equation proposed by investigators in the Chronic Kidney Disease Epidemiology (CKD-EPI) Collaboration (24). This equation was developed from a cohort of patients that included both healthy individuals and individuals with chronic kidney disease and that was much larger than the Modification of Diet in Renal Disease study. We preferred this equation because it is more accurate in subjects with GFR > 60 mL/min/1.73 m^2^, which our patients were expected to have given the inclusion criteria. Total serum insulin-like growth factor-1 (IGF-1) concentrations were determined by chemiluminescent immunoassay (Nichols Advantage, Nichols Institute Diagnostic, San Juan Capistrano, CA). High sensitivity C-reactive protein (hs-CRP) levels were measured by an automated instrument (CardioPhaseH hsCRP, Siemens, Italy).

### Cardio-Respiratory Monitoring

To confirm the diagnosis of OSAS, patients underwent a CRM during sleep (Compumedics, Somtè), with continuous non-invasive recordings of respiratory pressure and flow, thoracic-abdominal motion, oxygen saturation, ECG and body position. Results from the sleep studies were analyzed by a trained technician using the recommendations of the American Academy of Sleep Medicine (AASM) (25). The severity classification of OSAS was based on the apnea/hypopnea index (AHI): mild (5 < AHI < 15), moderate (15 < AHI < 30), and severe (AHI > 30). According with current clinical recommendations (22), patients with moderate or severe OSAS were prescribed with nocturnal CPAP with an automated device (ResMed AirSenseTM10 AutoSet/Elite- AutoSet TM CS-A). Moreover, all patients received counseling on sleep hygiene. CPAP has been titrated for three nights. The definitive value of CPAP is the amount of pressure that eliminates events in ~95% of the total sleep time (95th centile). Patients have been assessed monthly for compliance and effectiveness of the intervention. Compliance to the CPAP was measured using the data from the CPAP card; a patient was considered to be compliant when using CPAP at least 4 h/night (average) in at least 70% of nights. CPAP was considered effective if the AHI falled below 5/h.

### Blood Pressure Measurement

Blood pressure (BP) measurements readings of clinic BP were obtained in the non-dominant arm of the seated patient, after 5 min of quiet rest, with a mercury sphygmomanometer. Systolic and diastolic BP was recorded at the first appearance (phase I) and disappearance (phase V) of Korotkoff sounds. Baseline BP values were the average of the last two of the three consecutive measurements obtained at intervals of 3 min (26).

### Arterial Stiffness

These measurements were obtained by a validated system (Sphygmocor^TM^; AtCor Medical, Sydney, Australia) that employs high-fidelity applanation tonometry (Millar) and appropriate computer software for the analysis of pressure wave (Sphygmocor^TM^). Aortic pulse wave velocity (PWV) was determined from carotid and femoral pressure wave forms. Carotid to femoral transit time (DT) was computed from the foot-to-foot time difference between carotid and femoral waveforms. The distance between the surface markings of the sternal notch and femoral artery was used to estimate the path length between the carotid and femoral arteries (L), and PWV computed as L/DT.

### Statistical Analysis

The analysis was performed on 165 subjects, and results were expressed as mean ± SD or as percent frequency. Comparisons between groups were made by paired or unpaired Student *t*-test or χ^2^ test as appropriate. Patients were divided in two groups: OSAS + (AHI>15) and OSAS– (AHI < 5). Linear regression analysis was used to assess the relationship between baseline e-GFR and different covariates in all population and in the groups separately. Thus, the same analysis was performed in the OSAS+ to evaluate the major determinant of the e-GFR variation (Δe-GFR), before and after 6 months of CPAP, considering the variation (Δ) of the examined covariates. After this, a multivariate regression analysis was performed to determinate the independent predictor of the Δe-GFR. A value of *P* < 0.05 was considered statistically significant. All calculations were made with a standard statistical package (SPSS for Windows version 16.0).

## Results

**Figure 1:**
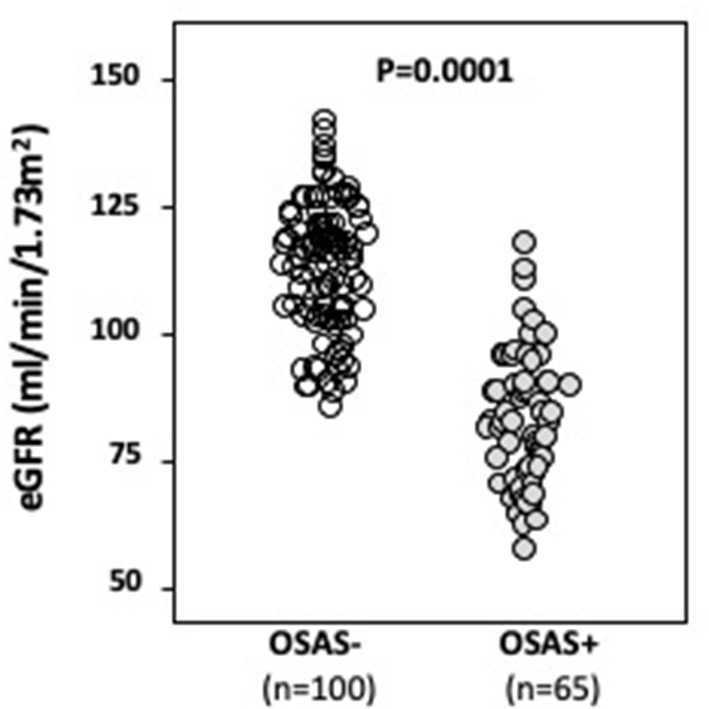
Mean e-GFR baseline values in OSAS– and OSAS+ groups. In this figure, we graphically reported mean baseline e-GFR values of both OSAS– and OSAS+ groups. As indicated, OSAS+ subjects showed statistically significant lower baseline e-GFR values when compared to OSAS– subjects.

From an initial cohort of 198 obese subjects, 33 were excluded because they were diagnosed with mild OSAS (5 < AHI <15); baseline anthropometric, clinical and humoral characteristics of these patients are described in the Supplementary Material. The remaining 165 patients were divided into two groups (OSAS– *n* = 100, and OSAS+ *n* = 65) on the basis of CRM results. Baseline anthropometric, clinical, and humoral characteristics of the whole study population and of the two groups separately are listed in Table 1. There were no significant differences between the two groups with respect to mean age, gender, BMI, waist, DBP, total and LDL-cholesterol. As expected, patients with OSAS showed significantly increased values of AHI, oxygen desaturation index (ODI), cumulative sleep time percentage with oxyhemoglobin saturation <90% (TC90), and NC; furthermore, OSAS+ patients also showed higher values of SBP, hs-CRP, UA, triglycerides, fasting plasma glucose, 2 h-postload plasma glucose, HOMA and PWV, while IGF-1 and HDL-cholesterol were significantly lower in this group. Of interest, OSAS+ patients showed significantly higher values of microalbuminuria (Table 1) and significantly lower e-GFR values (84.0 ± 13.1 vs. 114.5 ± 12.8 mg/dl/1.73 m^2^, *P* < 0.0001) compared with OSAS– group (Figure 1).

**Table 1:** Baseline anthropometric, clinical, and humoral characteristics of OSAS– and OSAS+ groups.

	**OSAS –**	**OSAS +**	***P***
	***n* = 100**	***n* = 65**	
Age, years	54.3, 10.9	56.2, 11.0	0.251
Gender, M/F	62/38	48/17	0.159
Current smokers, *n*	25	42	0.000
BMI, kg/m^2^	36.6, 3.3	36.9, 6.3	0.711
Waist, cm	114.8, 11.6	118.6, 14.1	0.059
NC, cm	44.1, 3.4	47.4, 3.2	0.0001
AHI	2.3, 1.4	42.2, 25.1	0.0001
ODI	7.5, 3.4	24.0, 11.6	0.0001
TC90, %	4.9, 2.8	21.1, 5.0	0.0001
SBP, mmHg	131.1, 15.2	140.0, 12.6	0.0001
DBP, mmHg	82.1, 10.4	81.7, 9.8	0.827
hs-CRP, mg/L	3.5, 2.4	6.5, 6.4	0.0001
Microalbuminuria, mg/dL	13.1, 3.6	22.7, 11.3	0.0001
UA, mg/dL	5.7, 1.4	6.5, 1.4	0.001
Total Chol, mg/dL	198.3, 23.5	200.0, 12.8	0.603
LDL-Chol, mg/dL	126.1, 23.3	129.4, 16.2	0.311
HDL-Chol, mg/dL	47.1, 10.5	42.3, 9.9	0.004
Triglycerides, mg/dL	125.8, 25.0	141.1, 25.0	0.0001
Fasting plasma glucose, mg/dL	98.1, 15.9	107.6, 12.7	0.0001
2 h-postload plasma glucose, mg/dL	152.6, 23.2	172.4, 25.8	0.023
Insulin, mU/L	15.7, 7.3	21.0, 3.0	0.0001
HOMA	3.8, 2.1	5.6, 1.0	0.0001
IGF-1, ng/mL	156.5, 28.1	141.6, 32.8	0.002
PWV, m/s	7.1, 1.0	7.4, 1.9	0.104

*AHI, apnea hypopnea index; BMI, body mass index; DBP, diastolic blood pressure; e-GFR, estimated glomerular filtration rate; HDL-Chol, high-density lipoprotein cholesterol; HOMA, homeostatic model assessment; hs-CRP, high-sensitivity C-reactive protein; IGF-1, insulin-like growth factor; LDL Chol, low-density lipoprotein cholesterol; NC, neck circumference; ODI, oxygen desaturation index; PWV, pulse wave velocity; SBP, systolic blood pressure; TC90, sleep time percentage with oxyhemoglobin saturation (SpO_2_) < 90%; Total Chol, total cholesterol; UA, uric acid*.

**Figure 2:**
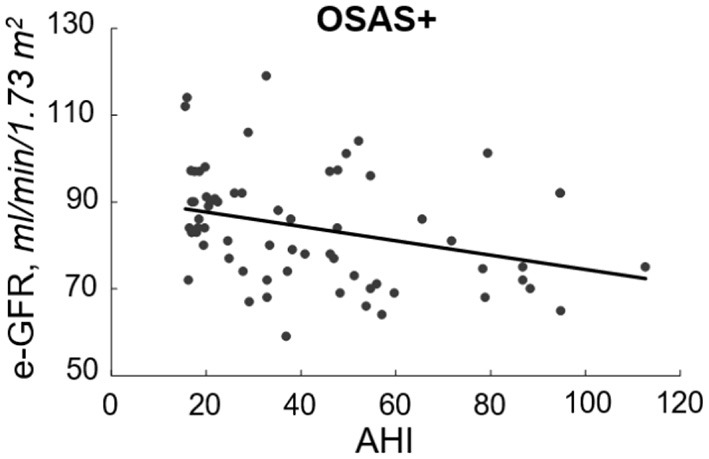
Inverse relationship between e-GFR and AHI in OSAS+ group. In this figure, we reported the inverse relationship between e-GFR and AHI in subjects with OSAS.

At linear regression analysis (Table 2) e-GFR was significantly and inversely associated with AHI (*r* = −0.667, *P* < 0.0001), ODI (−0.510, *P* < 0.0001), TC90 (−0.639, *P* < 0.0001), microalbuminuria (−0.457, *P* < 0.0001), SBP (−0.225, *P* = 0.002), UA (*r* = −0.208, *P* = 0.004), hs-CRP (*r* = −0.197, *P* = 0.006), triglycerides (*r* = −0.150, *P* = 0.027), while it was directly associated with HDL-cholesterol (*r* = 0.147, *P* = 0.029) and IGF-1 (*r* = 0.200, *P* = 0.005) in the whole study population. In OSAS– group e-GFR was significantly related to hs-CRP (*r* = 0.292, *P* = 0.002) and DBP (*r* = 0.189, *P* = 0.030), while it was inversely related to AHI (*r* = −0.297, *P* < 0.001). In OSAS+ patients, e-GFR was significantly and inversely related only with AHI (*r* = −0.319, *P* = 0.005) (Figure 2) and microalbuminuria (*r* = −0.217, *P* = 0.041).

**Table 2:** Univariate relationship between e-GFR and different covariates in the whole study population and in the two groups separately.

	**All**		**OSAS -**		**OSAS** **+**	
	***r***	***P***	***r***	***P***	***R***	***P***
AHI	−0.667	0.0001	−0.297	0.001	−0.319	0.005
ODI	−0.510	0.0001	0.069	0.249	0.113	0.185
TC90, %	−0.639	0.0001	0.122	0.113	0.198	0.057
HOMA	−0.332	0.0001	0.056	0.291	−0.030	0.406
Microalbuminuria, mg/dL	−0.457	0.0001	0.084	0.204	−0.217	0.041
SBP, mmHg	−0.225	0.002	0.096	0.172	−0.174	0.083
UA, mg/dL	−0.208	0.004	0.040	0.346	−0.076	0.273
IGF-1, ng/mL	0.200	0.005	0.001	0.496	0.075	0.275
hs-CRP, mg/L	−0.197	0.006	0.292	0.002	−0.046	0.357
Triglycerides, mg/dL	−0.150	0.027	0.095	0.172	0.129	0.153
HDL-Chol, mg/dL	0.147	0.029	−0.045	0.327	−0.013	0.458
DBP, mmHg	−0.088	0.129	0.189	0.030	−0.002	0.495
PWV, m/s	−0.081	0.149	0.013	0.450	0.033	0.396
BMI, kg/m^2^	−0.055	0.243	−0.023	0.411	−0.076	0.274
LDL-Chol, mg/dL	0.017	0.416	0.179	0.038	−0.008	0.474

*AHI, apnea hypopnea index; BMI, body mass index; DBP, diastolic blood pressure; HDL-Chol, high-density lipoprotein cholesterol; HOMA, homeostatic model assessment; hs-CRP, high-sensitivity C-reactive protein; IGF-1, insulin-like growth factor; LDL Chol, low-density lipoprotein cholesterol; ODI, oxygen desaturation index; PWV, pulse wave velocity; SBP, systolic blood pressure; TC90, sleep time percentage with oxyhemoglobin saturation (SpO_2_) < 90%; UA, uric acid*.

**Figure 3:**
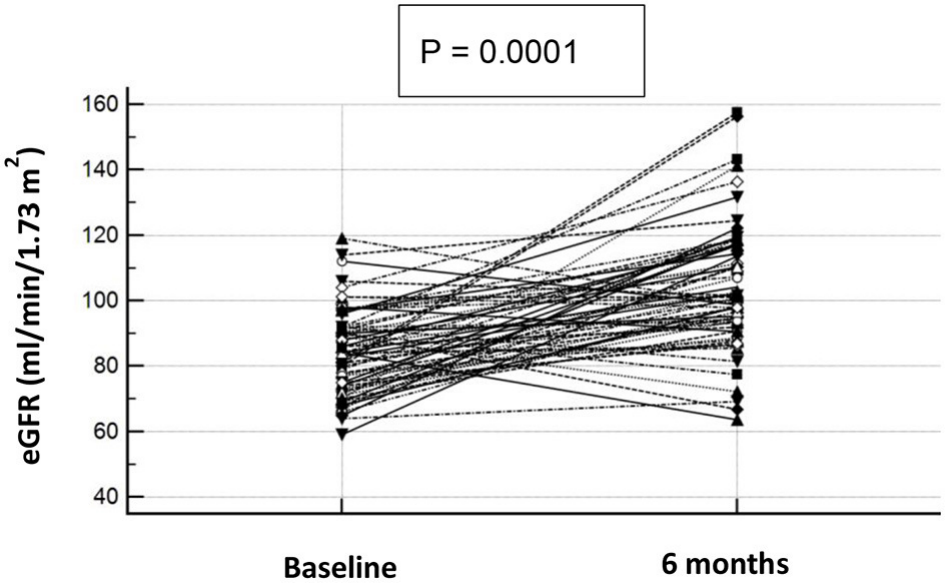
e-GFR baseline and after 6 months of CPAP therapy in OSAS+ subjects. Graphical representation of e-GFR variation after 6 months of CPAP therapy in OSAS+ subjects. Each dot represents a single subject.

Table 3 shows the clinical and biochemical characteristics of OSAS+ patients at baseline and after CPAP therapy. After 6 months, these subjects presented significantly lower values of BMI, waist, AHI, ODI, TC90, hs-CRP, UA, microalbuminuria, HOMA and PWV. Of interest, we observed a significant increase of e-GFR values (104.2 ± 19.0 vs. 84.0 ± 13.1 ml/min/1.73 m^2^, *P* < 0.0001) after 6 months of CPAP therapy (Figure 3); in two subjects in the OSAS+ group we detected an almost doubled value of e-GFR (from 80 to 160 ml/min/1.73 m^2^) at the end of the follow-up period, to be intended as a hyperfiltration due to a weight gain >5% from the basal value. Subsequently, in the same group, a linear regression analysis was performed between Δe-GFR, as dependent variable, Δ of different covariates and baseline e-GFR. As shown in Table 4, Δe-GFR was significantly and inversely correlated with ΔAHI (*r* = −0.482, *P* < 0.0001) and e-GFR at baseline (*r* = −0.543, *P* = 0.0001). Then, we performed a multiple regression analysis (Table 5) including (Model A) and excluding (Model B) baseline e-GFR from the model. We observed that Δe-GFR was influenced by e-GFR at baseline, that explained 28.4% of its variation, and by ΔAHI that determined another 9.5% of its variation. In the second model, ΔAHI resulted the main independent predictor of Δe-GFR explaining 22% of its variation, followed by ΔTC90 that explained another 5.1% of Δe-GFR variation.

**Table 3:** Anthropometric, hemodynamic and humoral characteristics of OSAS+ patients at baseline and after 6 months of CPAP therapy.

	**Baseline (*n* = 65)**	**T6 (*n* = 65)**	***P***
BMI, kg/m^2^	36.9, 6.3	36.5, 6.6	0.111
Waist, cm	118.8, 14.1	118.0, 14.7	0.018
AHI	42.2, 25.1	4.9, 2.9	0.0001
ODI	24.0, 11.6	6.5, 3.3	0.0001
TC 90, %	21.1, 5.0	16.6, 5.9	0.0001
SBP, mmHg	140.0, 12.6	135.7, 15.2	0.053
DBP, mmHg	81.7, 9.8	80.9, 9.7	0.612
hs-CRP, mg/L	6.5, 6.4	3.1, 2.0	0.0001
Microalbuminuria, mg/dL	22.7, 11.3	14.1, 6.3	0.0001
UA, mg/dL	6.5, 1.4	5.9, 1.4	0.014
HOMA	5.6, 1.0	3.8, 1.4	0.0001
IGF-1, ng/mL	141.6, 32.8	158.3, 27.4	0.003
PWV, m/s	7.4, 1.9	6.7, 1.4	0.034

*AHI, apnea hypopnea index; BMI, body mass index; DBP, diastolic blood pressure; e-GFR, estimated glomerular filtration rate; HOMA, homeostatic model assessment; hs-CRP, high-sensitivity C-reactive protein; IGF-1, insulin-like growth factor; ODI, oxygen desaturation index; PWV, pulse wave velocity; SBP, systolic blood pressure; TC90, sleep time percentage with oxyhemoglobin saturation (SpO_2_) < 90%; UA, uric acid*.

**Table 4:** Univariate relationship between Δe-GFR and different covariates in OSAS+ patients.

	***r***	***P***
ΔAHI	−0.482	0.0001
Baseline e-GFR, ml/min/1.73 m^2^	−0.543	0.0001
ΔTC 90, %	0.195	0.060
ΔMicroalbuminuria, mg/dL	−0.168	0.090
ΔTriglycerides, mg/dL	0.139	0.135
ΔODI	0.127	0.157
Δhs-CRP, mg/L	−0.104	0.205
ΔIGF-1, ng/mL	−0.067	0.298
ΔLDL-Chol, mg/dL	−0.048	0.352
ΔBMI, kg/m^2^	−0.024	0.425
ΔHOMA	0.006	0.480

*AHI, apnea hypopnea index; BMI, body mass index; e-GFR, estimated glomerular filtration rate; HOMA, homeostatic model assessment; hs-CRP, high-sensitivity C-reactive protein; IGF-1, insulin-like growth factor; LDL Chol, low-density lipoprotein cholesterol; ODI, oxygen desaturation index; TC90, sleep time percentage with oxyhemoglobin saturation (SpO_2_) < 90%*.

**Table 5:** Multiple linear regression models for Δe-GFR with (model A) and without (model B) baseline e-GFR.

	**Partial *r*^**2**^ (%)**	**Total *r*^**2**^ (%)**	***P***
**Model A**			
Baseline e-GFR, ml/min/1.73 m^2^		28.4	0.0001
ΔAHI	9.5	37.9	0.002
**Model B**			
ΔAHI		22.0	0.0001
ΔTC90, %	5.1	27.1	0.023

*AHI, apnea hypopnea index; e-GFR, estimated glomerular filtration rate*.

## Discussion

The most important finding obtained in this study, conducted in a population of obese patients with normal renal function, is that those with moderate-to-severe OSAS showed lower—even if in the normal range—baseline e-GFR values compared to subjects without OSAS; moreover, 6 months treatment with CPAP was effective in improve e-GFR by 20 ml/min/1.73 m^2^. Clinically relevant, ΔAHI resulted the most important independent predictor of Δe-GFR, allowing to affirm that the improvement of nocturnal respiratory function is the main determinant of e-GFR increase in this particular setting of patients. In fact, ΔAHI -or the resolution of apneas during CPAP-, and ΔTC90 -or the improvement of oxyhemoglobin saturation during sleep-, represent the main predictors of Δe-GFR, independently from metabolic, hormonal, and inflammatory status. These findings are consistent with other published literature, which identifies the resolution of chronic intermittent hypoxic episodes during sleep as the major pathophysiological mechanism of the improvement of endothelial glomerular function (10, 27, 28). Our data are in agreement with previous literature but it appears evident that there are other pathophysiological OSAS-related mechanisms responsible of renal function impairment in this setting of patients. Our hypothesis is that arterial stiffness, in addition to the inflammatory status characterizing obese patients, may negatively influence intraglomerular hemodynamic. In fact, arterial stiffness represents an independent predictor of cardiovascular, cerebral and renal morbidity (29); mineral metabolism disturbances, vascular calcifications, formation of advanced glycation end-products, and acute and chronic volume overload, occurring in ESRD and CKD patients, have been proposed as the main pathogenetic mechanisms linking arterial stiffness to renal disease (30). In our study population, there were not significant differences in PWV (a surrogate marker of arterial stiffness) between OSAS– and OSAS+ patients, despite significantly higher SBP values in the last group. Of clinical importance, PWV was significantly lower after 6 months of CPAP therapy, leading to hypothesize a positive impact of reduced arterial stiffness on renal hemodynamic. Moreover, it is well-established that CPAP therapy reduces CV risk in OSAS patients (19, 31) through an improvement in the insulin-resistant status. In addition, in a group of subjects affected by OSAS and CKD stage III-IV, CPAP therapy was effective in reducing proteinuria and increasing e-GFR after a year of treatment (32). Other studies have demonstrated that CPAP therapy reduces glomerular hyperfiltration with a consequent increase of renal plasma flow in obese OSAS patients (33). With this regard, it is important to remark that also cigarette smoking negatively impacts on renal function (34, 35); in our study population, in the OSAS+ group we observed a greater prevalence of current smokers than in the OSAS– subjects, contributing to the impairment of e-GFR in these subjects.

The novelty of our study is that, differently from other Authors, we considered a group of obese subjects with preserved renal function. Many studies describe a major prevalence of breathing sleep disorders in subjects affected by ESRD in comparison with those with normal renal function (36, 37). On the other hand, in the last few years, some Authors focused their attention on the prevalence of OSAS in the first stages of renal dysfunction, even if the majority of the studies is based on patients' history collection and not on sleep instrumental study (36). It is well-known that obesity, a risk factor of OSAS, is associated with glomerular hyperfiltration but, on the contrary, very little is known about the impact of breathing sleep disorders on renal hemodynamic in these subjects (20, 21, 37–39).

In conclusion, even if this study has several limitations such as the single-center non-blinded design and the small sample size, our data confirm previously published literature about the detrimental effect of OSAS on renal function also in subjects free from kidney disease; this detrimental effect may be reverted, at least in part, by CPAP therapy.

## Data Availability Statement

The raw data supporting the conclusions of this article will be made available by the authors, without undue reservation.

## Ethics Statement

The studies involving human participants were reviewed and approved by Comitato Etico Calabria Centro. The patients/participants provided their written informed consent to participate in this study.

## Author Contributions

MP, FP, and RM: Conceptualization; PS and AS: methodology; RM and PS: software; MP, GS, and FP: validation; RM: formal analysis; PS, BC, LM, MV, and ES: investigation; MP, RM, and PS: writing—original draft preparation; MP and FP: writing—review and editing; MP, AS, and FP: supervision. All authors have read and agreed to the published version of the manuscript.

## Conflict of Interest

The authors declare that the research was conducted in the absence of any commercial or financial relationships that could be construed as a potential conflict of interest.
