# Supplementary material for: Continuous Positive Airway Pressure Improves Renal Function in Obese Patients With Obstructive Sleep Apnea Syndrome
Source: Front Med (Lausanne). 2021 Mar 3;8:642086. doi: 10.3389/fmed.2021.642086 (PMC7965975; doi:10.3389/fmed.2021.642086)
Supplement: Supplementary file 1 [file Data_Sheet_1.PDF]

Baseline anthropometric, clinical and humoral characteristics of mild OSAS patients

	Mild OSAS<br>N = 33
**Age,** ***years***	60±10.5
**Gender,** ***M/F***	24/13
**Current smokers,** ***n***	67
**BMI,** ***Kg/m²***	31.7±4.9
**Waist,** ***cm***	106±9.9
**NC,** ***cm***	40±3.3
**AHI**	10±3.7
**ODI**	19.5±11.6
**TC90,** ***%***	10.2±17.1
**SBP,** ***mmHg***	128±14.8
**DBP,** ***mmHg***	78±10.8
**hs-CRP,** ***mg/L***	5.7±7.3
**Microalbuminuria,** ***mg/dL***	19.9±17.4
**UA,** ***mg/dL***	5.9±2
**Total Chol,** ***mg/dL***	174.6±40.6
**LDL-Chol,** ***mg/dL***	110.3±36.4
**HDL-Chol,** ***mg/dL***	47.3±13.6
**Triglycerides,** ***mg/dL***	133.5±25.0
**e-GFR,** ***ml/min/1.73 m²***	97.8±30.6
**Fasting plasma glucose,** ***mg/dL***	117.4±37.7
**Insulin,** ***mU/L***	19±9.8
**HOMA**	2.3±3.1
**IGF-1,** ***ng/mL***	149.1±30.5
**PWV,** ***m/sec***	8.9±1.4

AHI= apnea hypopnea index, BMI= body mass index, DBP= diastolic blood pressure, e-GFR= estimated glomerular filtration rate, HDL-Chol= high-density lipoprotein cholesterol, HOMA= homeostatic model assessment, hs-CRP= high-sensitivity C-reactive protein, IGF-1= insulin-like growth factor, LDL Chol= low-density lipoprotein cholesterol, NC= neck circumference, ODI= oxygen desaturation index, PWV= pulse wave velocity, SBP=systolic blood pressure, TC90= sleep time percentage with oxyhemoglobin saturation (SpO_2) < 90%, Total Chol= total cholesterol, UA = uric acid.
